# Cases of Lungworm in Cats from Southern Poland in the Autopsy and Cytological Material

**DOI:** 10.3390/pathogens14070630

**Published:** 2025-06-25

**Authors:** Stanisław Dzimira, Małgorzata Kandefer-Gola, Rafał Ciaputa, Marta Demkowska-Kutrzepa

**Affiliations:** 1 Department of Pathology, Faculty of Veterinary Medicine, Wroclaw University of Environmental and Life Sciences, Norwida Str. 31, 50-375 Wroclaw, Poland; malgorzata.kandefer-gola@upwr.edu.pl (M.K.-G.); rafal.ciaputa@upwr.edu.pl (R.C.); 2 Department of Parasitology and Fish Diseases, Faculty of Veterinary Medicine, University of Life Sciences in Lublin, Akademicka Str. 12, 20-950 Lublin, Poland; marta.demkowska@up.lublin.pl

**Keywords:** lung nematodes, domestic cats, necropsy, cytology, bronchoalveolar lavage

## Abstract

Lungworms in carnivorous domestic animals are infestations that are relatively uncommon. However, in felines, especially wild ones, they are not at all rare. This study aimed to assess the prevalence of respiratory parasite infections (lung nematodes) in domestic cats based on necropsy and cytological examinations and to highlight the cytological examination of respiratory material as a practical and straightforward diagnostic method. For the presence of lung parasites, necropsy material (cadavers of cats) and samples submitted for cytological examinations from 2005 to 2022 were analyzed. In total, 730 cat samples from southern and southwestern Poland were examined—420 autopsied and 310 cats whose samples were examined cytologically. The material was collected using the bronchoalveolar lavage (BAL) and submitted for cytological examination. Out of 420 cat autopsies, larvae and eggs of *Aelurostrongylus abstrusus* were found in 4 individuals (0.95%). In cytological material obtained from BAL, out of 310 samples analyzed, larvae and eggs of *A. abstrusus* and *Capillaria aerophila* were found in only 2 cases (0.64%). Respiratory parasitic infections in cats can pose a serious health risk, especially with high intensity, in young animals. Considering that such cases present a diagnostic challenge, it is advisable to encourage cat owners to limit their pets’ contact with intermediate and paratenic hosts and use anthelmintics to combat lung parasites.

## 1. Introduction

Lung nematodes in carnivorous domestic animals are infestations that are not very common, even though feline lung nematodes are not rare in wild animals. In Europe, domestic cats are most frequently infected with *Capillaria aerophila* (syn. *Euceoleus aerophilus*) (Creplin, 1839) and *Aelurostrongylus abstrusus* (Railliet, 1898) [1,2,3]. The former inhabits the lungs, bronchi, trachea, nasal cavities, and head sinuses. The latter, which is less frequently encountered, occurs in the bronchi and pulmonary alveoli. These parasites have been observed in Poland in free-living animals (foxes, lynxes) and less frequently in domestic animals such as dogs and cats [4,5,6,7]. Reports of lung nematode infestations are increasingly frequent in European and global literature. Among feline lung nematodes, the developmental cycle of *A. abstrusus* is the best understood. It follows a heteroxenous cycle, with intermediate hosts including both shell-less and shelled snails (snail-borne disease) from the genera *Agriolimax, Limax, Helix, Chondrula, Helicella, Monacha, Epiphragmophora*, and *Helminthoglypta* [8]. Within three weeks, the larvae undergo two molts (first-stage larvae → second-stage larvae → third-stage larvae: *L*1 *→ L*2 *→ L*3) inside the snail, after which they reach the third, infective stage, which carnivorous animals then ingest. The ingested larvae penetrate the mucosa of the esophagus, stomach, or anterior sections of the small intestine, from where they migrate via the lymphatic or hematogenous route to the lungs. There, they reach sexual maturity, and the females lay eggs approximately 33–76 days (4–8 weeks) after infection. The patent period of infestation can last 5–6 years [9]. First-stage larvae, *L*1 (with a characteristically S-shaped tail), hatch and migrate up the trachea to the larynx, are coughed up, swallowed, and excreted with feces after 14 days [10]. The spread of this infestation may involve paratenic hosts, such as vertebrates that feed on snails, including some amphibians, reptiles, mice and other small rodents, and birds (sparrows, chicks of domestic birds like chickens and ducks) [10,11]. Considering the dietary preferences of the final hosts—carnivorous animals—the role of paratenic hosts is significant for completing the life cycle and spreading the parasite. By consuming infected snails, these animals accumulate the invasive larvae (*L*3) of the parasite, becoming the primary source of infestation, including cats. Nematodes of the species *C. aerophila* have low host specificity. This is one of the most common lung nematode infestations in dogs, cats, and wild predators [10,12,13]. They are a potentially zoonotic agent capable of causing severe lung disease by residing in the submucosa of the trachea, bronchi, and bronchioles [10]. The developmental cycle of *C. aerophila* is direct. Adult females lay eggs with characteristic bipolar plugs (60–70 × 35–40 μm), which are passively transported via the bronchial tree to the trachea and pharynx, where they are swallowed. After passing through the digestive tract, the eggs reach the environment via feces, where, after an incubation period of approximately 5–6 weeks, the larvae inside the egg casings reach the invasive *L*1 stage. The earthworm is a paratenic host in the *C. aerophila* cycle, increasing the parasite’s invasive potential. Infection of the final host occurs via ingestion. The first-stage larvae released from the egg casings migrate to the lungs through the circulatory and lymphatic systems. The prepatent period lasts approximately 25–29 days, while the patent period lasts 10–11 months [9]. Felines also exhibit mixed infestations caused by respiratory system nematodes such as *Troglostrongylus brevior* and *Oslerus rostratus* [1,14]. Due to the morphological similarity of *L*1 larval stages present in feces to *A. abstrusus*, these infestations were likely misidentified in the past. The spread of these nematodes by wild animals cannot be ruled out [13].

Respiratory parasite infections manifest primarily through respiratory symptoms, ranging from subclinical infections to life-threatening conditions. Lungworms in cats can cause a wide range of symptoms, including coughing, trouble breathing, open-mouth breathing, or panting, sneezing, wheezing, loss of appetite (anorexia), weight loss and lethargy. In extreme cases of long-term infection, severe invasions of tiny worms can be seen in what your cat has coughed up [6].

This study aims to determine the frequency of respiratory parasite infections in domestic cats based on necropsy and cytological examinations and to highlight the cytological examination of respiratory material as a simple and practical diagnostic method.

## 2. Materials and Methods

### 2.1. Study Area and Sampling

**Figure 1:**
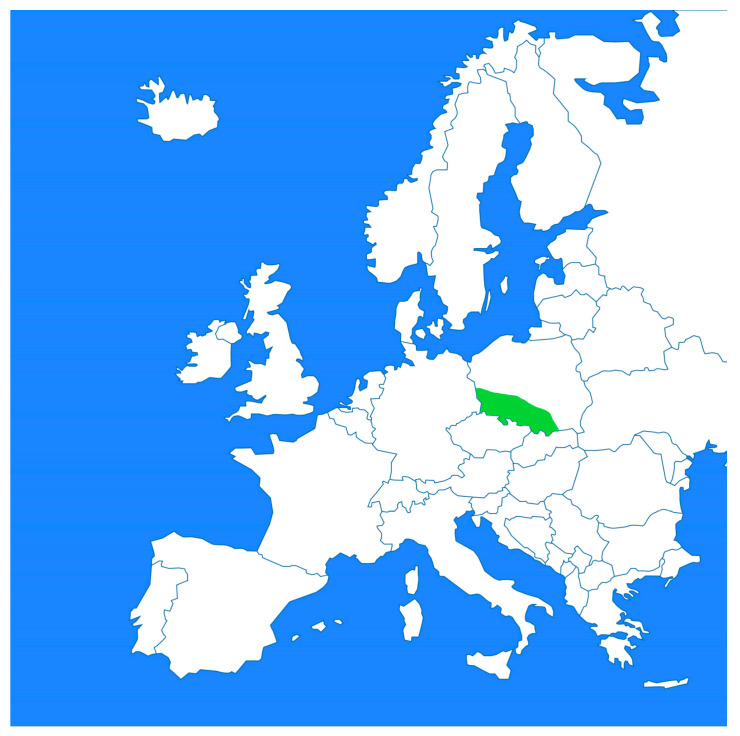
Map of Europe showing the approximate area of southern and southwestern Poland from which the research material originated (green).

For the presence of lung parasites, necropsy material (cadavers of cats subjected to autopsy) and samples submitted for cytological examinations from 2005 to 2022 were analyzed. A total of 730 samples from cats from southern and southwestern Poland were examined. The approximate area of origin of the material is shown in Figure 1, marked in green. The causes of death were analyzed based on necropsy results of 420 cats autopsied at the Department of Pathology of the University of Environmental and Life Sciences in Wrocław from 2005 to 2022. The cats submitted for necropsy were aged between 6 weeks and 17 years, of both sexes, spayed and unspayed, indoor, outdoor, stray, and of unknown status (corpses found by random people). They belonged to various breeds—Maine Coon, Sphynx, Neva Masquerade, Bengal, Russian Blue, Persian, Siamese, British Blue, Devon Rex, Norwegian Forest, and dominant mixed breeds. The second group consisted of 310 cats whose samples were examined cytologically. The material was collected using the bronchoalveolar lavage (BAL) method and submitted for cytological examination. Private veterinarians submitted the material in slides from fluid sediment obtained via BAL for evaluation. BAL was included in the diagnostic panel for cats with chronic conditions that are resistant to treatment, characterized by persistent coughing and dyspnea. The material for cytological study was obtained from cats aged 6 months to 14 years, of both sexes, including spayed and unspayed individuals, primarily from home-grown cats, although some cases involved material from shelter cats. Crossbreed cats dominated this material, although preparations from pedigree cats (as above) also occurred. The bronchoalveolar lavage test was performed at the request of a physician. We do not know whether other tests, such as stool tests, were performed simultaneously in these patients.

### 2.2. Laboratory Analysis

Histopathological examination was performed on necropsy samples of lung tissue taken from different affected lobes. The material was preserved in 10% buffered formalin, embedded in paraffin blocks, sectioned into 4 µm slices, and stained using the standard hematoxylin and eosin method. Histopathological slides and smears from the obtained sediment were fixed, stained with the routine hematoxylin and eosin method, and examined under a bright-field microscope (Olympus BX53, Tokyo, Japan) equipped with an Olympus UC90 camera. The measurements were taken using cellSens Standard V.1 software (Olympus, Tokyo, Japan). The parasitic infection was expressed as the prevalence of infection (number of cats infected with parasites/total number of examined cats × 100%).

## 3. Results

Out of 420 cat autopsies, respiratory system parasites were found in 4 individuals (0.95%). These cases involved free-living animals (stray cats) only fed by humans during winter. These cats had not undergone preventive treatments like deworming, vaccination, or flea and tick control. Autopsy examinations of these cats, aged approximately 7 to 12 years and of both sexes, non-spayed (1 male, and 3 female), and of mixed breed, revealed severe emaciation, mild jaundice of the mucous membranes and subcutaneous tissue, and an empty gastrointestinal tract with signs of catarrhal inflammation in the stomach and small intestines. The lungs were highly irregularly congested, with a surface that was uneven, finely granular, and contained multiple nodules ranging in size from a pinhead to a pea, sometimes merging into larger, grayish-brown and grayish-yellow, firm foci that slightly protruded above the surface. These lesions macroscopically resembled pulmonary tumors. The trachea and bronchial tree contained varying amounts of thick, mucous secretions.

**Figure 2:**
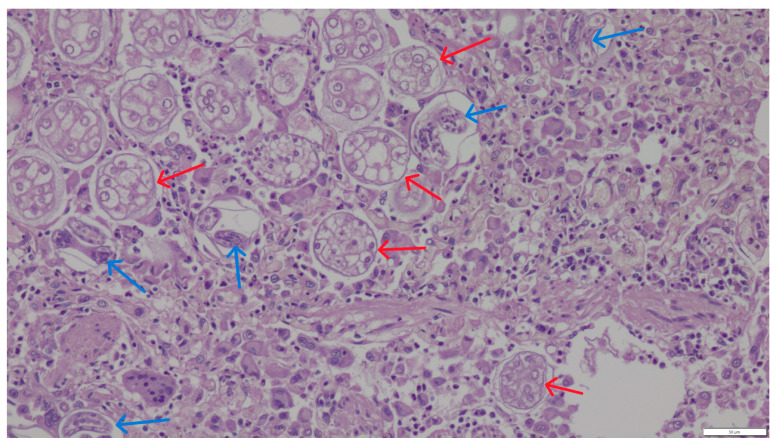
First-stage larvae (blue arrows) and eggs (red arrows) of *A. abstrusus* in pulmonary alveoli. HE staining, magnification 200×.

**Figure 3:**
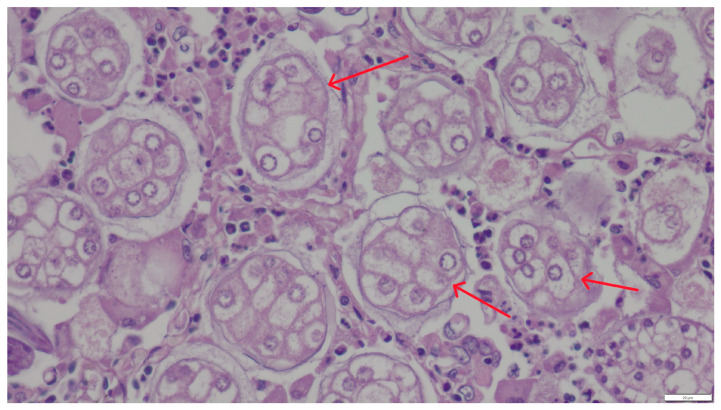
Numerous *A. abstrusus* eggs (red arrows) in pulmonary alveoli. HE staining, magnification 400×.

Microscopic examination of the lung samples from both cats revealed numerous first-stage larvae and *A. abstrusus* eggs located within the alveoli and small bronchioles, sometimes filling their lumen (Figure 2 and Figure 3). A severe inflammatory response of lymphocytes, histiocytes, plasma cells, and eosinophils surrounded the affected alveoli and bronchioles. The stroma exhibited proliferation of interstitial connective tissue. The walls of blood vessels had significantly thickened, indicating the chronic nature of the disease. The mucus sampled from the bronchial tree contained individual, elongated larvae measuring 356–380 μm in length and 16–24 μm in width, tapered at both ends. The head was conical and rounded, while the tail was undulating, equipped with a slight, bluntly-ended dorsal projection characteristic of *Aelurostrongylus* larvae. The parasite morphology and larval measurements corresponded to descriptions provided by other authors [11].

**Figure 4:**
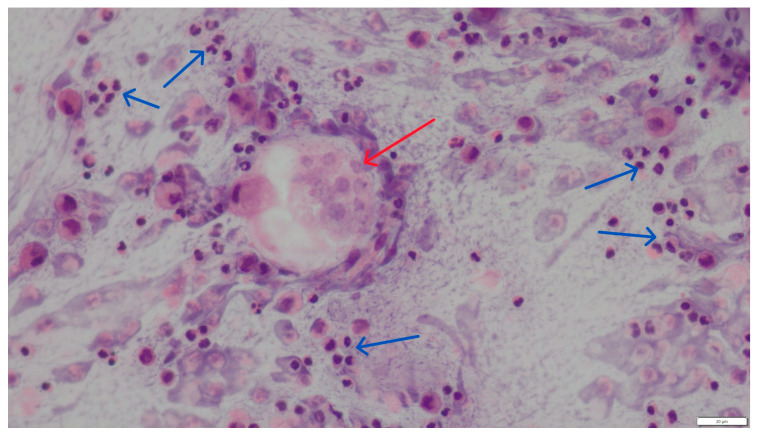
Sediment smear from lavage fluid, inflammatory infiltrate cells (blue arrows), and a sporulating egg (red arrow). HE staining, magnification 400×.

**Figure 5:**
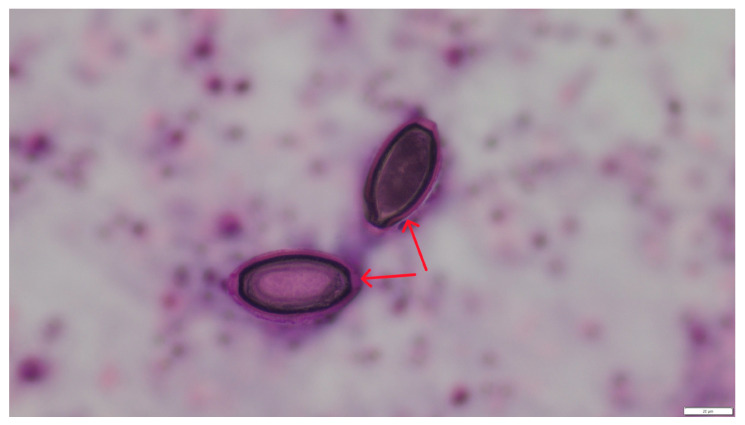
A sediment smear from lavage fluid and background showing inflammatory infiltrate cells in mucus masses and *C. aerophila* eggs (red arrows). HE staining, magnification 400×.

**Figure 6:**
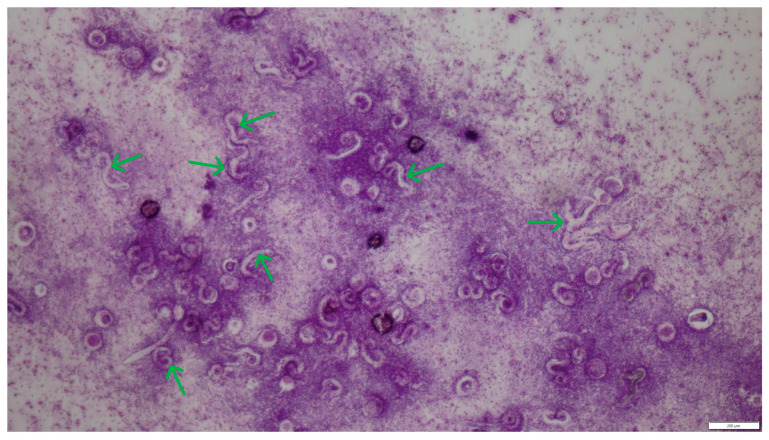
Sediment smear from lavage fluid, inflammatory infiltrate cells in mucus masses, and numerous *C. aerophila* larvae (green arrows). HE staining, magnification 40×.

In cytological material obtained from bronchial-alveolar lavage out of 310 samples analyzed, larvae and eggs of respiratory parasites were found in only 2 cases (0.64%). Morphometric analysis of the parasite developmental stages, based on literature data [11], allowed the identification of *A. abstrusus* and *C. aerophila* infections. The two infected cats were male, 6 months old and -7 years old, respectively, both not spayed. These two diagnosed cases were significantly different. The first was a six-month-old indoor male cat diagnosed with chronic respiratory failure and inconclusive radiographic findings (widespread lung opacities). The second was a seven-year-old male cat admitted to an animal shelter, where a chronic, untreatable cough prompted the BAL examination. Cytological preparations from both patients showed weakly basophilic mucus masses, numerous neutrophils, eosinophils, and macrophages (Figure 4). Within the mucus, numerous larvae and individual eggs at various stages of development were observed between inflammatory infiltrate cells (Figure 5 and Figure 6). The eggs measured approximately 60 μm in length and 30 μm in width.

## 4. Discussion

In Europe, parasite species found in the respiratory system of cats include *Aelurostrongylus abstrusus*, *Angiostrongylus chabaudi*, *Troglostrongylus brevior*, *Troglostrongylus subcrenatus*, and *Capillaria aerophila*. Among the aforementioned species, the dominant ones in both global and Polish literature are *C. aerophila* (syn. *Euceoleus aerophilus*) (Creplin, 1839) and *A. abstrusus* (Railliet, 1898) [1,2,3]. The first documented case of *A. abstrusus* infection in a domestic cat in Poland was described by Dzimira and Popiołek [4], with the first antemortem diagnosis reported by Studzińska et al. [6]. In wild lynxes, *A. abstrusus* infections were identified through fecal analysis by Szczęsna et al. [5]. In studies by Wierzbowska et al. on the carcasses of free-living cats in southern Poland, only *C. aerophila* cases were identified [7]. This parasite was more common in younger cats (1–2 years old), regardless of their habitat.

A retrospective study of 716 cat fecal samples from southeastern Poland revealed *A. abstrusus* infections in 7 individuals [15]. Literature data indicate that lungworms of the *Aelurostrongylus* genus are increasingly reported in cats across Europe and, together with *C. aerophila*, are now considered the most significant respiratory parasites of felines [1,10]. According to research by Giannelli et al. [1], which analyzed lungworm infections in cats across 12 European countries (Austria, Belgium, Bulgaria, France, Greece, Hungary, Italy, Portugal, Romania, Spain, Switzerland, and the UK) over one year (March 2015–February 2016), *A. abstrusus* infections were found in 210 of 1990 tested samples (10.6%). These cases varied in frequency, from single occurrences (e.g., Belgium and Switzerland—1 each, France—4, Spain—5) to higher numbers (e.g., Hungary—27, Bulgaria—33, Italy—44). In the same study, *C. aerophila* was significantly rarer, with only 31 total cases, from isolated instances (Belgium, France, Italy) to more frequent detections (Greece—5, Romania—8, Bulgaria—13). Other data from Italy report the prevalence of *A. abstrusus* infection at a roughly similar level, 17.3% and 18.5%, depending on the region of the country where the examined fecal samples originated [12]. In a study conducted by Romanian scientists, *A. abstrusus* was found in 5.6% of domestic cats and *C. aerophila* in 3.1% of the studied population (out of 414 faecal samples) [16]. Quite numerous cases of aelurostrongylosis in cats in Germany were described over a decade ago by Barutzki et al. [17]. Among the data collected from examining 391 cats, they identified 24 positive cases, i.e., 6.6%. The highest prevalence of infection was recorded in the southwest of Germany (12 positive cases). In northern Germany, Becker et al. demonstrated the presence of aelurostrongylosis in only 1% of the tested cats, with 8 positive cases out of 837 cats tested by the feces flotation method [18]. In Hungary, a group of 303 cats was examined, of which 60 were infected with *A. abstrusus*, constituting 19.8% [19]. In Albania, *A. abstrusus* was found in as many as 50% of examined cats, while *C. aerophila* was detected in 16.7% [20]. In a study conducted by Danish authors on 147 cats, including 125 free-living and 22 domestic cats, *A. abstrusus* infection was detected in 13.6% of the examined population [21]. Studies of outgoing cats’ feces in Sweden revealed only 1 positive case of *A. abstrusus* out of 205 tested attempts [22]. Among stray cats in northwestern Portugal, *A. abstrusus* infection was found at a level of 17.4% [19]. Other studies from Portugal showed the occurrence of this parasite in homeless city cats at 12.4%, and *C. aerophila* at 0.6%. [23]. In Estonia, Tull et al. tested 290 cats from the shelter and did not find cases of aelurostrongylosis; however, they did find 6 (2.1%) infections of *C. aerophila* [24]. Based on the above data, it can be concluded that lungworm infections in cats are more prevalent in areas with higher average daily temperatures. Europe is mainly located in the temperate climate zone. There is a subpolar climate in the northern reaches—northern Iceland, the extreme ends of Norway, and northwestern Russia. In the northeastern part of the continent, it is cold, while in the rest, it is warm (maritime, continental, and transitional). The southern parts of Europe, including the peninsulas of Iberia, Apennine, and Balkan, as well as the islands of the Mediterranean Sea, are located in the zone of subtropical climates, which are maritime in many areas and continental in Bulgaria and central Spain. More cases of lungworm in cats have been reported in areas approximately below 50° north latitude (the latitude where Paris is located) than in areas located above this geographical coordinate. This thesis appears to be confirmed by the data cited above, particularly the multi-center study by Giannelli et al., which describes the situation in 12 European countries. Our results confirm this—the area from which the studied material comes lies north of the above-mentioned latitude. However, this thesis is contradicted by the results of Salling Olsen et al. [21], who, in an even more northerly area, found that the prevalence of parasite infection was only slightly lower than that reported by Italian or Portuguese authors [12,25].

Respiratory parasites in domestic cats are relatively uncommon in other parts of the world. Lopez-Osorio et al. [3] detected *A. abstrusus* in Colombia in only 2 of 473 fecal samples (0.4%). In the USA, retrospective analysis of over 3.5 million commercial laboratory tests using zinc sulfate flotation revealed positive results in 4721 cases (0.13%), with *Baermann* method testing yielding 75 positive cases out of 3625 samples (2.06%) [26]. However, using fecal analysis and autopsy, Willard et al. found an 18.5% infection rate (20 out of 108 cats) in a shelter population in Alabama [27]. Gerdin et al. identified *A. abstrusus* infections as a cause of circulatory failure and anesthesia-related sudden death in 9 of 54 examined cases in New York between 2009 and 2010 [2]. Respiratory parasite infections manifest primarily through respiratory symptoms, ranging from subclinical infections to life-threatening conditions [6,28]. In young or weakened animals and cases of heavy infestations, symptoms may include exercise intolerance, heart failure, and circulatory issues [29,30,31,32]. Most *A. abstrusus* or *C. aerophila* infections are diagnosed incidentally in free-living cats that were never, or only occasionally, dewormed by caregivers. Among domestic cats that had access to the outdoors and potential intermediate/paratenic hosts but were dewormed regularly, *A. abstrusus* infections were significantly less frequent, both antemortem (cytology, fecal analysis) and postmortem (autopsy). According to the literature, this infection is most often observed from October to March and in ‘outdoor’ cats up to 6 months of age suffering from respiratory inflammation symptoms [15]. Respiratory helminthiasis is often not included in the differential diagnosis. However, advanced treatment-resistant respiratory inflammation, persistent coughing, and progressive emaciation should warrant BAL analysis for first-stage larvae [6,33]. It should be remembered, however, that BAL may be useful in certain clinical contexts, but should not replace copromicroscopic examination as a first-line diagnostic tool. The usefulness of BAL is somewhat limited by difficulties in morphological imaging, especially for the inexperienced clinician.

Seasonal larval shedding peaks in winter months makes fecal Baermann testing a reliable diagnostic method [6,26,31,34]. Also, stool examination using larvoscopic diagnostics, e.g., the Baermann method, effectively detects lungworm larvae [6,15,18,23,28]. However, it should be remembered that due to the long prepatent period of infection, tests based on the search for *A. abstrusus* larvae may give false negative results. Another problem may be the seasonality of L1 larvae excretion, resulting in their most intensive release into the environment from October to March. An additional difficulty is that the Baermann method takes 24 h, and after this time, larvae of other nematodes may also be present in the preparation. A lower-sensitivity in vivo method, also used in diagnosing respiratory capillariasis, is flotation using solutions with high specific gravity (e.g., saturated NaCl and MgCl2 solutions). In this case, the test’s sensitivity is enhanced by using active flotation and centrifugation [35]. In the context of coproscopic diagnostics, stool collected directly from the rectum should be examined regardless of the method used. When taking a sample from the environment, there is a risk of contamination of the sample with dispersive forms of saprophytic nematodes. Serological tests (ELISA), detecting parasite antigens or antibodies circulating in the blood, are characterized by high sensitivity in diagnosing pulmonary parasitoses. However, it should be remembered that these tests, despite their high sensitivity and specificity, carry a risk of false negative results in the case of low-intensity infection [36]. Molecular tests are diagnostic techniques that also provide a high probability of correct diagnosis of infection. PCR methods are increasingly used in scientific research, but in standard veterinary practice, their use is limited by their high price and long determination time. It should be assumed that between 2005 and 2010, phylogenetic and sequencing methods were not commonly used, but these would have increased the study’s reliability. Additionally, advanced imaging, serological, and molecular techniques may enhance diagnostic accuracy [21,22,24].

Table 1 presents data on the intensity of lungworm cases in European countries over the last twenty years chronologically.

Given the increasing number of reported cases, the impact of climate change on parasite distribution should be considered. Warmer conditions favor parasite larvae’s survival and their range expansion. Increased populations of intermediate hosts, urbanization, and improved diagnostic tools also contribute to better detection rates. Regular deworming and preventive treatments for domestic and free-living cats remain essential in controlling respiratory helminth infections. Although the extent of *Metastrongyloidae* infection in cats in Poland is low and similar to that described in the northeastern part of Germany, it can be assumed that such cases will become more frequent [15]. This is related to climate change, since warming creates favorable conditions for the spread of the infection. This will promote the shift of the boundary of more frequent occurrences of the described infections above the line of countries where such cases are more numerous (Italy, Greece, Albania, Bulgaria, Hungary, Romania). This is already evident in the reported cases in Central and Northern European countries. Transitional weather conditions characterize the temperate climate in Poland. In southern Poland, it is warmer than in the north due to the greater amount of solar energy that reaches it. In the area marked in Figure 1, average monthly temperatures are approximately 1–2 °C higher than in the northern and northeastern parts of Poland. This factor favors the development and survival of intermediate hosts and, thus, invasive stages of parasites. It should be noted that the increasing population of shell-less snails in recent years may have a significant impact. They serve as a reservoir for larvae and allow them to survive unfavorable weather conditions (L1 larvae can survive in the environment for about 14 days). The ongoing urbanization of suburban areas and increased populations of intermediate and paratenic hosts are also not without significance. On the other hand, the awareness of pet owners, their care for their animals, and the diagnostic and therapeutic capabilities of veterinarians have improved. The latter now have access to complex, systemic preparations that protect animals from external parasites such as fleas and eliminate endoparasites.

## 5. Conclusions

The presented studies are retrospective. The number of lungworm infections is relatively small, 4/420 in autopsy cases and 2/310 in cytology (BAL). However, it is essential to remember that parasitic infections of the respiratory system in cats can pose a serious health risk, especially at high intensity, in young animals. Considering that such cases pose a diagnostic challenge, it is worth encouraging cat owners to limit contact of their pets with intermediate and paratenic hosts, and to use anthelmintics that are also effective in combating lungworms.

A limitation of the study is that lung biopsies were not collected for histopathological examination from every necropsied cat. If the cause of death was determined (e.g., locomotor injury, poisoning, post-traumatic or post-operative hemorrhage, etc.), no histopathological examination of the lungs was performed.

The role of cytological examination is not the first instance, but the gold standard test is the copromicroscopic examination with the Baermann technique.

## Figures and Tables

**Table 1:** Cases of lungworm (*A. abstrusus*—A or *C. aerophila*—C or combined—CO) in domestic and stray cats in European countries in the last 20 years (chronologically, selected data).

No.	Country	Study Methods/Techniques	Number of Cats in Study	Number and Percent of Positive Cats	References
1	Poland (southwestern region)	Autopsy/histopathology	1 (case report)	1/(100%) A	Dzimira et al., 2005 [4]
2	Italy (central and southern regions)	floatations with sugar and zinc sulphate solutions and a Baermann technique	227	8/(17.3%) A 12/(18.5%) A	Traversa et al., 2008 [12]
3	Portugal	Baermann-Wetzel method	97	17/(17.4%) A	Payo-Puente et al. 2008 [25]
4	Romania	Sodium chloride flotation	414	23/(5.6%) A 13/(3.1%) C	Mircean et al. 2010 [16]
5	Albania	Autopsy/histopathology	18	9/(50%) A 3/(16.7%) C	Knaus et al., 2011 [20]
6	Germany	zinc chloride/sodium chloride flotation and Baermann technique	391	24/(6.6%) A	Barutzki et al., 2012 [17]
7	Germany	Baermann-Wetzel method	837	8/(1%) A	Becker et al., 2012 [18]
8	Portugal	Flotation and sedimentation techniques	162	(12.4%) A (0.6%) C	Waap et al., 2014 [23]
9	Denmark	modified Baermann technique	147	17/(13.6%) A	Salling Olsen et al., 2015 [21]
10	Poland (southeastern region)	flotation, decantation technique, and Baermann’s larvoscopic method.	716	7/(0.98%) A	Szczepaniak et al., 2017 [15]
11	12 European countriess (Austria, Belgium, Bulgaria, France, Greece, Hungary, Portugal, Romania, Spain, Italy, Switzerland, Great Britain)	The McMaster technique and a quantitative Baermann-Wetzel method	1990	210/(10.6%) CO	Gianelli et al., 2017 [1]
12	Sweden	Baermann’s larvoscopic method.	205	1/(0.48%) A	Grandi et al., 2017 [22]
13	Hungary	Baermann-Wetzel method	303	60/(19.8%) A	Kiszely et al., 2019 [19]
14	Poland (southern region)	McMaster technique	81	2/(2.47%) C	Wierzbowska et al., 2020 [7]
15	Estonia	flotation technique	290	6/(2.1%) C	Tull et al., 2021 [24]
16	Poland (south and southwestern region)	Autopsy/histopathology	420	4/(0.95%) A	Our study
17	Poland (south and southwestern region)	Cytology (BAL)	310	2/(0.64%) CO	Our study

## Data Availability

The original contributions presented in this study are included in the article. Further inquiries can be directed to the corresponding author.

## Abbreviations

The following abbreviations are used in this manuscript:

BAL	Bronchoalveoral lavage
